# Assessment of the Feasibility and Acceptability of Using Water Pasteurization Indicators to Increase Access to Safe Drinking Water in the Peruvian Amazon

**DOI:** 10.4269/ajtmh.18-0963

**Published:** 2020-05-04

**Authors:** Kristen Heitzinger, Stephen E. Hawes, Claudio A. Rocha, Carlos Alvarez, Carlton A. Evans

**Affiliations:** 1 Innovacion Por la Salud Y el Desarollo (IPSYD), Asociación Benéfica Prisma, Lima, Peru;; 2 IFHAD: Innovation for Health and Development, Department of Infectious Disease, Imperial College London, London, United Kingdom;; 3 Department of Epidemiology, University of Washington, Seattle, Washington;; 4 U.S. Naval Medical Research Unit No. 6, Lima, Peru;; 5 Regional Center for Disease Prevention and Control, Loreto Regional Ministry of Health, Iquitos, Peru;; 6 IFHAD: Innovation for Health and Development, Laboratory of Research and Development, Universidad Peruana Cayetano Heredia, Lima, Peru

## Abstract

Approximately two billion people lack access to microbiologically safe drinking water globally. Boiling is the most popular household water treatment method and significantly reduces diarrheal disease, but is often practiced inconsistently or ineffectively. The use of low-cost technologies to improve boiling is one approach with potential for increasing access to safe drinking water. We conducted household trials to evaluate the feasibility and acceptability of water pasteurization indicators (WAPIs) in the Peruvian Amazon in 2015. A total of 28 randomly selected households were enrolled from a rural and a peri-urban community. All households trialed two WAPI designs, each for a 2-week period. Ninety-six percent of participants demonstrated the correct use of the WAPIs at the end of each trial, and 88% expressed satisfaction with both WAPI models. Ease of use, short treatment time, knowledge of the association between WAPI use and improved health, and the taste of treated water were among the key factors that influenced acceptability. Ease of use was the key factor that influenced design preference. Participants in both communities preferred a WAPI with a plastic box that floated on the water’s surface compared with a WAPI with a wire that was dipped into the pot of drinking water while it was heating (77% versus 15%, *P* < 0.001); we selected the box design for a subsequent randomized trial of this intervention. The high feasibility and acceptability of the WAPIs in this study suggest that these interventions have potential to increase access to safe water in resource-limited settings.

## INTRODUCTION

Globally, an estimated 2.1 billion people lack access to safely managed water,^1^ and 502,000 deaths annually are attributed to unsafe or insufficient drinking water in low- and middle-income countries (LMICs).^2^ Household water treatment (HWT) has been shown to be effective in decreasing diarrheal disease associated with poor drinking water quality^3,4^ and may be the only feasible way to improve drinking water quality in areas where piped water infrastructure is difficult to build or maintain. However, motivating adoption and sustained use of HWT technologies has been a major challenge to achieving long-term improvements in household drinking water quality and reductions in diarrheal disease.^5–8^ Boiling is practiced by approximately 70% of HWT users in LMICs and is by far the most popular HWT method.^9^ It has generally been associated with significant improvements in drinking water quality^10–19^ and with a 42% reduction in diarrheal disease risk.^20^ Consistent and effective practice of boiling is, however, limited by the risk of recontamination,^17,21,22^ time,^23^ cost of fuel,^24^ risk of injury or scalding,^25,26^ and its contribution to indoor air pollution.^27^

In light of the challenges of scaling up the use of commercial HWT technologies, some have proposed that the use of interventions to improve and expand boiling could be an effective approach to achieve health gains associated with access to safe household drinking water.^20,28^ One such intervention is a water pasteurization indicator (WAPI), a reusable thermosensitive tool that indicates to users when water has been heated to the temperature of pasteurization (65°C). This sub-boiling temperature is sufficient for pathogen inactivation and can reduce the time, cost, and contribution to indoor air pollution associated with the heat treatment of water. Since the development of the WAPI in 1992, several different models have been manufactured, and kits to produce one model ($0.42 per WAPI) are sold by a chapter of Rotary International. Research to evaluate the impact of WAPI use on household drinking water quality and diarrheal disease has yielded conflicting results,^29,30^ and a better understanding of the factors that influence WAPI adoption is needed to inform the design of effective interventions. In a study in rural Kenya, 29% of households continued to pasteurize their water 4 years after implementation of a WAPI intervention, indicating that the intervention was highly acceptable, but factors affecting the feasibility or acceptability of the intervention were not assessed.^29^ A randomized trial of household drinking water quality interventions in Peru demonstrated the feasibility of teaching rural households to use a WAPI.^30^ Participants in this trial most frequently cited benefits of WAPI use to be fuel saving, time saving, and ease of use, and most frequently cited disadvantages to be the need to monitor the WAPI to use it correctly and difficulty of use.^30^

In the Peruvian Amazon, 77% of households report boiling their household drinking water,^31^ yet approximately 87% of households with a child younger than 5 years drink water that is microbiologically contaminated,^32^ which suggests that boiling practice or water storage practices are not optimal. To inform the design of a WAPI intervention for a future health impact trial among households with children younger than 5 years, we investigated the factors affecting the feasibility and acceptability of WAPI designs in rural and peri-urban communities near Iquitos in the Peruvian Amazon.

## MATERIALS AND METHODS

### Study site.

We conducted this study in the health post catchment areas of two communities of the Loreto region in northeastern Peru between March and May 2015. One community, Ollanta Humala, is a peri-urban shantytown on the outskirts of the city of Iquitos; the other, Varillal, is a rural community approximately 15 km southwest of Iquitos. In the peri-urban community, most households purchased their drinking water from private water vendors who sold water daily from trucks that circulated in the community; some had covered wells in their yards. In the rural community, households accessed drinking water from a combination of piped household connections, community standpipes (shallow wells), covered shallow wells in the home, and ravines in the area. Households using improved water sources were known to store their household drinking water because of the distance to the source or inconsistent provision of water in the case of piped household connections and thus could benefit from household treatment. The quality of vended drinking water was also unknown at the start of the study. Liquid chlorine was distributed free of charge by the regional Ministry of Health at local health posts, and bleach was inexpensive and commercially available. Barriers to the adoption of water chlorination in a peri-urban community near Ollanta Humala have been described.^33^

### Study design.

We evaluated two WAPI designs using randomized crossover trials of improved practice (TIP) study design. Trials of improved practice is a formative research method used to assess the feasibility and acceptability of a health-related behavior.^34^ The TIP method, which is also known as household trials, entails repeated visits to participant homes to test new behaviors and assessments of whether the participant tried the new behavior and their reaction to it.^34^ This method has been used to inform the design of other household-level health-related interventions^35–39^ and has been used previously in the Amazonian region.^40^ We defined feasibility as whether participants could be taught to use the WAPI model correctly and whether the WAPI could be routinely used. We defined acceptability as participants’ satisfaction with use of the models.

**Figure 1.**
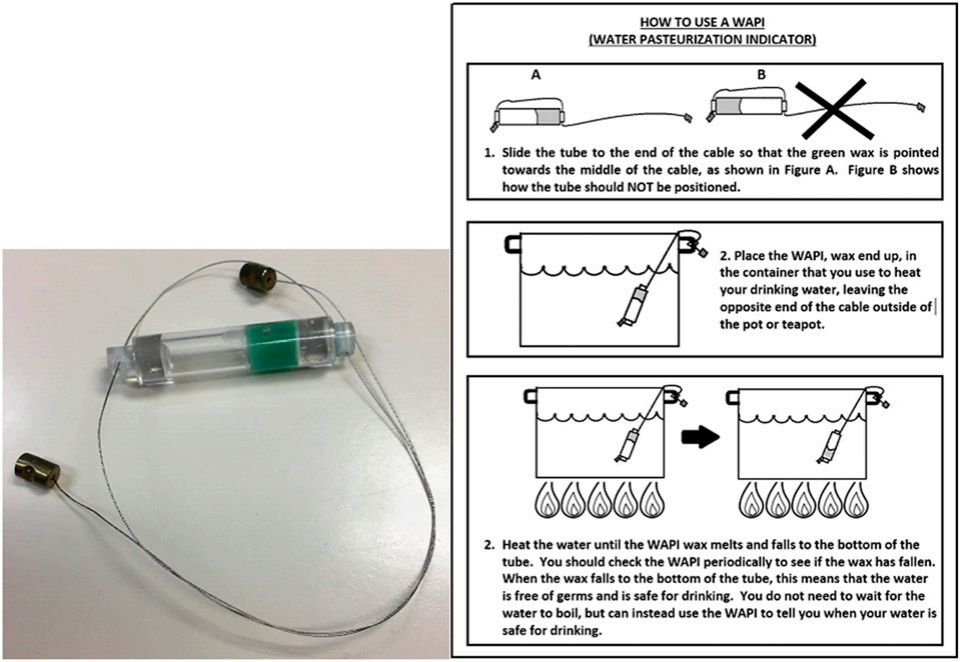
Wire model of water pasteurization indicator (Solar Cookers International) with pictorial instructions for use. Image reproduced with permission. This figure appears in color at www.ajtmh.org.

**Figure 2.**
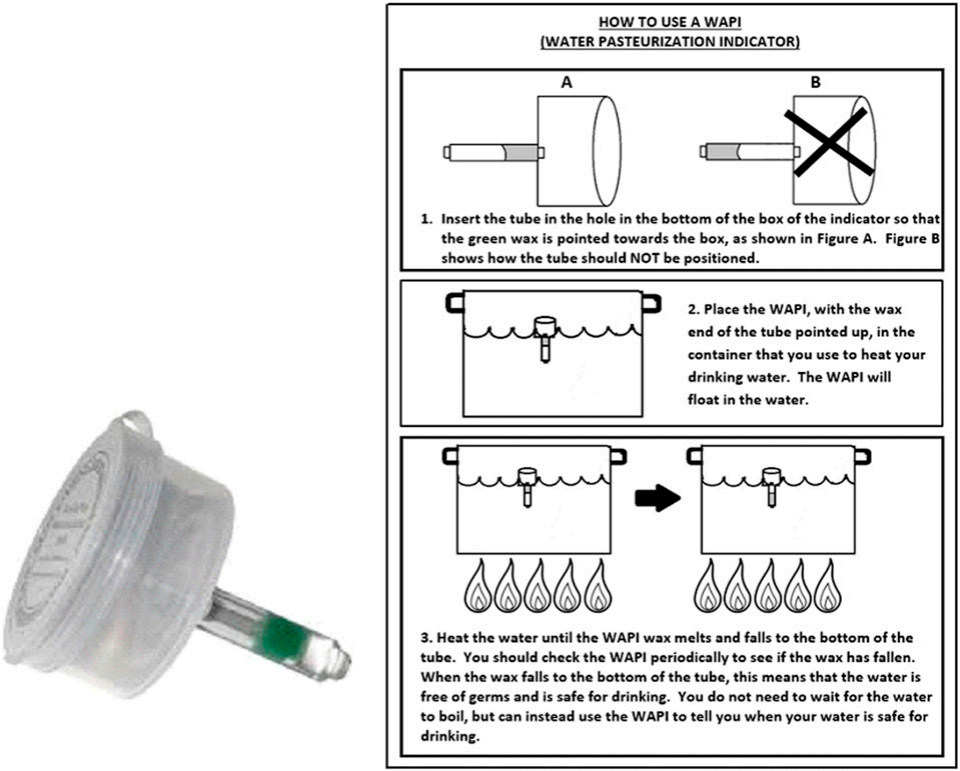
Box model of water pasteurization indicator (Sun Ovens International) with pictorial instructions for use. Image reproduced with permission. This figure appears in color at www.ajtmh.org.

In both communities, we obtained data from the early childhood health program of the health posts to create a list of eligible households containing at least one child younger than 5 years. We selected households from the list using a random number generator and continued enrollment until a total of 28 participants was reached (14 in each community). The adult (≥ 18 years) primarily responsible for the management of the household drinking water supply in each eligible household was invited to participate. Trained field workers conducted a semi-structured interview with participants at the enrollment visit to assess demographic characteristics, household water sources, and other information relevant to water treatment, sanitation, and hygiene practices. In each community, households were randomly assigned to first use either a “wire model” or a “box model” of the WAPI for a 2-week trial period (Figures 1 and 2). These two models were selected for the trials because they were of low cost (< $5) and could potentially be locally produced.

All households received written and pictorial instructions on how to use each WAPI model and were instructed to use the WAPI to treat their drinking water, but were not given specific instruction regarding the timing of treatment. A field worker demonstrated the use of the model and requested that the participant demonstrate correct use of the model during the enrollment visit. Instruction continued until the participant demonstrated correct use of their assigned model. The wire model consisted of a 2-cm polycarbonate tube containing a wax that melted at the temperature of water pasteurization (Solar Cookers International, Sacramento, CA; Figure 1). A stainless steel cable connected to the tube allowed users to dip or hang the WAPI in a pot of drinking water while it was heating to determine whether the water had been heated sufficiently to be free of microbiological contamination. Field-workers instructed participants to place the wire model in the boiling pot such that the wax end of the tube pointed upward. They instructed participants to remove the wire model from the boiling pot once the wax had fallen to the bottom of the tube, which indicated that the water had been pasteurized and was safe to drink. Field workers instructed participants to hang the WAPI from a nail or other object until the wax hardened and the WAPI could be stored. The box model consisted of a small plastic box with a circular hole in the bottom and a top that snapped in place (Sun Ovens International, Elburn, IL; Figure 2). To use the box model, participants inserted an accompanying polycarbonate tube in the hole in the bottom of the box in such a position that the wax end was at the top of the tube. They then dropped the box model in the water, where it would float on the water’s surface. Field workers instructed users to lift the box out of the water periodically to verify whether the wax had fallen to the bottom of the tube. Field workers instructed users to remove the box model from the water at this time and turn it upside down on a clean surface until the wax hardened. Users were instructed to then remove the tube from the bottom of the box model and store it inside the box until the next use. Field staff instructed the participants to store their WAPI inside the plastic covering of their instructions or leave it hanging from a nail near the place where they heated their water. Participants were instructed to store the box model in a safe place near where they heated their water. No special instruction regarding avoidance of recontamination of pasteurized water or water handling or storage was given to participants; if participants recontaminated their pasteurized water in the process of removing the WAPI from the heating container, a significant reduction in the number of any pathogenic organisms introduced was still expected because inactivation of bacteria, viruses, and protozoa does occur at or below the temperature of water pasteurization.^41^ Instruction in using the assigned model was not repeated after the participant was trained. The polycarbonate tubing used in both WAPIs was made of FDA-compliant resins thus is considered safe for heating and direct contact with drinking water; bisphenol A (BPA) leaching and health risks from use of WAPIs were not a concern.

Field workers conducted weekly semi-structured household interviews to assess knowledge, use, and perceptions of the WAPI models. Two weeks following the enrollment visit, field workers collected the WAPIs that had been initially assigned to participants and replaced them with the other model and its corresponding paper instructions. Field-workers trained participants in the use of the second model in the same way as was done at the enrollment visit. Two weeks after the second WAPI was distributed to households, field workers assessed participants’ model preference and distributed toiletries to thank the households for their participation. Participants did not retain either model at the end of the survey. Household source and stored water samples were collected in 100-mL sterile Whirl-Pak bags (Nasco, Fort Atkinson, WI) at the enrollment visit and at the end of each 2-week trial. Samples were transported in coolers containing ice packs to the study center generally within 6 hours and analyzed for most probable number of *Escherichia coli* using the Compartment Bag Test (Aquagenx, Chapel Hill, NC), as per manufacturer’s instructions.^42^ Samples were incubated at 35–44.5°C for a minimum of 20 hours, and positive and negative controls were incubated with the water samples every day to confirm that the tests were functioning properly. All household interviews were audio-recorded.

### Data analysis.

Study staff transcribed recorded responses to open-ended survey questions and thematically coded them line by line. They used interview notes to supplement the transcripts, which were also thematically coded. Key themes were sorted by community and by indicator model and categorized using the integrated behavioral model for water, sanitation, and hygiene interventions (IBM-WASH).^43^ Qualitative data were managed and analyzed using ATLAS.ti version 8.2.30 (ATLAS.ti Scientific Software Development GmbH, Berlin, Germany). We identified contextual, psychosocial, and technology factors that influenced the feasibility and acceptability of the WAPI designs at each level of the IBM-WASH framework. Data were analyzed for the overall study population and separately by the type of water source (improved or unimproved) and water treatment practices at enrollment (no treatment, treatment with chlorine or bleach, and boiling) to assess differences in results by those key characteristics. Quantitative survey data and water sample data were entered in Access databases and exported to STATA 14.2 (StataCorp, College Station, TX) for descriptive analysis. Cross-tabulations were used for descriptive analysis. The prevalence of *E. coli* contamination in paired source and stored water samples was compared using a McNemar’s test. The 2017 World Health Organization/United Nations Children’s Fund (WHO/UNICEF) Joint Monitoring Programme definitions were used to classify drinking water sources as improved or unimproved.^44^

### Ethical considerations.

This study was approved by the Institutional Review Boards of Asociación Benéfica Prisma, the University of Washington, the U.S. Naval Medical Research Unit No. 6, and the Loreto Regional Ministry of Health. All study participants provided written informed consent before the initiation of any study procedures.

## RESULTS

Of the 28 participants, 93% were homemakers and 64% had completed elementary school (Table 1). At enrollment, nearly all (12/14) of the participants in the peri-urban community reported using water delivered by a tanker truck or cart, with water drums as their primary drinking water source; in the rural community, households most frequently used a piped source (7/14), another improved source (1/14), tanker truck water (3/14), or an unimproved source (3/14) (Table 1). Forty-six percent (13/28) of participants reported that they did not treat their household drinking water at baseline, 39% (11/28) reported using chlorine or bleach, and 14% (4/28) reported boiling. Approximately half (47%; 7/15) of the participants who treated their household drinking water reported having treated the water stored in the home at the time of the enrollment visit. Household drinking water treatment practices and stored drinking water quality did not differ significantly between the two sites (Table 1).

**Table 1:** Demographic and water, sanitation, and hygiene characteristics of study participants by community, Peruvian Amazon, 2015

	Total (*N* = 28), *n* (%)		Rural (N = 14), *n* (%)		Peri-urban (*N* = 14), *n* (%)		*P*-value
Median age (years), range*	32 (18–67)		31 (19–63)		32 (18–67)		0.82
Female	28	(100.0)	14	(100.0)	14	(100.0)	Could not be calculated
Education†							0.28
Less than primary School	10	(35.7)	7	(50.0)	3	(21.4)	
Completed primary School	12	(42.9)	4	(28.6)	8	(57.1)	
Completed secondary School or more education	6	(21.4)	3	(21.4)	3	(21.4)	
Occupation†							> 0.99
Homemaker	26	(92.9)	13	(92.9)	13	(92.9)	
Independently employed	2	(7.1)	1	(7.1)	1	(7.1)	
Mean household size, range‡	7 (3–15)		5 (3–7)		8 (3–15)		0.02
Primary water source†							< 0.001
Delivered water	15	(53.6)	3	(21.4)	12	(92.9)	
Piped household connection	3	(10.7)	3	(21.4)	0	(0.0)	
Community standpipe	4	(14.3)	4	(28.6)	0	(0.0)	
Other improved source	3	(10.7)	1	(7.1)	2	(7.1)	
Unimproved source	3	(10.7)	3	(21.4)	0	(0.0)	
Any household water treatment	15	(53.6)	8	(57.1)	7	(50.0)	0.71
Chlorine/bleach	11	(39.3)	6	(42.9)	5	(35.7)	0.70
Boiling†	4	(14.3)	2	(14.3)	2	(14.3)	> 0.99
Treated water present in home†	7	(25.0)	5	(35.7)	2	(14.3)	0.39
*E. coli* detected in stored water†	17	(68.0)	8	(61.5)	9	(75.0)	0.67

* Wilcoxon–Mann–Whitney test.

† Fisher’s Exact test.

‡ Student’s *t*-test.

At enrollment, participants in both communities frequently cited health, particularly children’s health, and avoidance of disease as factors that motivated household drinking water treatment. Participants noted the lack of availability of treatment materials, particularly chlorine at the health post, as a key barrier to household treatment. In contrast with rural participants, peri-urban participants frequently noted the belief that their water source was treated and safe as a reason for nontreatment of household drinking water. Peri-urban participants reported buying 1 to 4 gallons at a time, depending on the household size, and using that water over 2 days. At both sites, participants frequently stored their household drinking water in a pot on the stove or in a large plastic container.

### Water pasteurization indicator feasibility and use.

Nearly all participants correctly demonstrated how to use a WAPI at the end of each 2-week trial (26/27 participants for the first trial and 25/26 for the second). One household lost their WAPI during the first trial and therefore could not complete the demonstration; the same household refused to complete the WAPI demonstration at the end of the second trial. The tube of one of the wire models cracked before the end of the 2-week trial and could no longer be used; no other WAPIs had structural problems during the study. During the household trials, two households from the peri-urban community were lost to follow-up. Ninety-five percent (106/112) of planned follow-up visits were completed. During the follow-up period, two rural households, neither of which were treating their water at enrollment, reported not using the WAPIs; one of these reported not using the wire model and cited the use of a treated drinking water source as the reason for nonuse, whereas the other (the household that did not complete the WAPI demonstrations) reported not using either model and cited a lack of habit of treating household drinking water. During the follow-up period, 53% (27/51) of stored water samples were contaminated with *E. coli.* There was no significant difference in the prevalence of *E. coli* contamination in stored drinking water by the assigned WAPI model (*P* = 0.12). In the peri-urban community, only seven source water samples were collected during follow-up, precluding a meaningful comparison of the prevalence of *E. coli* contamination in source and stored water samples. In the rural community, the prevalence of contamination in paired source and stored samples was similar (*P* = 0.76).

### Contextual factors.

At the societal/structural level, the regional Ministry of Health had a policy to provide liquid chlorine free of charge at local health posts, although, as noted by some participants at the enrollment visit, chlorine was not consistently available. At the community level, bleach was inexpensive and commercially available. Participants frequently compared WAPI use with water treatment using chlorine or bleach, suggesting that these two contextual factors contributed to prior experience using chlorine or bleach products and thus affected WAPI acceptability. Participants who reported treating their household drinking water with chlorine or bleach at enrollment most frequently compared WAPI use with this prior experience. By contrast, participants who reported boiling or nontreatment of their household drinking water most commonly compared WAPI use with boiling, although some participants did compare WAPI use with treatment with chlorine or bleach. Participants frequently noted that using a WAPI to treat their household drinking water took less time than using chlorine or bleach and made water treatment easier. Households that reported use of chlorine or bleach at enrollment very frequently noted that the taste of WAPI-treated water compared favorably with that of water treated with chlorine or bleach, whereas households that did not treat their household drinking water at enrollment had both positive and negative perceptions of the taste of WAPI-treated water and households that boiled rarely mentioned the taste of WAPI-treated water. Thus, the perceived disadvantages of chlorine products relative to the WAPIs may have contributed to its acceptability, particularly among households using chlorine or bleach. As one participant mentioned,

In comparison, I used to treat with chlorine and that took some time and had a taste. Now that I am treating with the indicator, it seems good to me and it doesn’t have any taste at all and is easy and fast. [Peri-urban woman, age 31, purchased drinking water from cart with small drums]

In the peri-urban community, nearly all of the households purchased water from small trucks, which resulted in an expenditure of approximately three soles ($0.91) every 2 days for a large (e.g., 10 people) household and half of that for a smaller (five person) household. One peri-urban participant reported changing from using primarily vended water to using rainwater, her secondary water source, because she felt she could ensure the safety of her drinking water using the WAPI without the necessity of purchasing potentially higher quality drinking water.

At the household level, gender roles and responsibilities were primary factors influencing the feasibility of consistent WAPI use. Women were the primary WAPI users because they were the household member most often at home, responsible for cooking, and were mothers and caretakers of other household members in this setting. In the minority of households in which WAPI use was a shared responsibility or used primarily by a household member other than the study participant, the primary WAPI user was generally a woman with more time to use the WAPI. Neighbors were typically not aware of the participant’s WAPI use, and interpersonal relationships outside the home had limited influence on WAPI use.

At the individual level, nearly all of the participants were homemakers and had frequent opportunities to use the WAPI. Participants commonly reported using the WAPI in the morning as a part of their typical duties, suggesting that it was feasible to integrate repeated use into their routine. However, low literacy was a factor that prevented WAPI use for some participants. One participant with less than a primary school education withdrew from the study after the enrollment visit because of a lack of confidence in her ability to successfully complete the trials, and one participant delegated WAPI use to a literate family member she perceived as more capable of using it correctly. She explained why her daughter was the primary WAPI user in her household:

She reads more. With me, I don’t read. How am I going to do that (use the WAPI)? [Peri-urban woman, age 63, purchased drinking water from cart with small drums]

### Psychosocial factors.

At the interpersonal/household level, some participants noted that use of the WAPIs helped them to protect the health and well-being of their children and families, which contributed to the acceptability of use. As one participant noted,

I like [the WAPI] because it’s very good for my health and my family’s health. It helps us to be healthier, to drink water without any worry because we know that it is good, healthy. [Peri-urban woman, age 25, purchased drinking water from cart with small drums]

At the individual level, knowledge of the relationship between transmission of waterborne pathogens and risk to the health of their children and families were factors that contributed to the acceptability of the WAPI models.

Participants noted greater self-efficacy using the WAPI than using chlorine or bleach because the WAPI could not be incorrectly dosed, and the wax dropping to the bottom of the tube provided a visual sign that the water had been correctly treated. As one participant stated:

[The WAPI] makes treatment easier…than using bleach…because with bleach sometimes you can’t measure it well, sometimes you use a bit too much and it can cause us harm…too much bleach burns the stomach, or it can cause diarrhea. [Peri-urban woman, age 25, purchased drinking water from cart with small drums]

At the habitual level, the expectation that household drinking water was safer after treatment was a factor that motivated WAPI use. One participant said,

The water that we collect sometimes has microbes in it. Sometimes they can cause us harm….the water is safer if it is treated with the indicator. [Rural woman, age 63, used household piped drinking water source]

### Technology factors.

At the household level, there was disagreement regarding the effect of WAPI use on the smell and taste of household drinking water, and reactions of children and other household members to the taste of the WAPI-treated water influenced the acceptability of WAPI use. Although some older household members reported that the taste of WAPI-treated water compared favorably with that of either boiled or chlorinated water, some children expressed a dislike for the smoky smell and taste of WAPI-treated water. One participant reported using the WAPI-treated water to make lemonade and thereby improve the taste for her children. Some households reported that a certain habituation period was needed for their children to become accustomed to the taste of water treated with the WAPIs or other methods, and that the person responsible for water treatment had a role in encouraging their children to drink treated water. One participant explained:

When I used to boil water, [my family] wouldn’t drink it, but I treat [the water] with [the WAPI] now, and all of them drink…They didn’t like it at the beginning, but this [water] doesn’t taste like smoke. All of them drink it. [Peri-urban woman, age 45, purchased drinking water from cart with small drums]

None of the households that had negative perceptions of the smell or taste of WAPI-treated water reported having stopped using the intervention.

At the individual level, the taste of the treated water was also an important factor influencing the acceptability of the WAPIs. Participants had generally positive perceptions of the taste of the WAPI-treated water. They reported that it had no taste, had the taste of “pure” untreated water, or that its taste was preferable to that of chlorinated or boiled water. As one participant reported:

If it hadn’t been for this [WAPI], I wouldn’t have treated my water…I’ve treated it with chlorine [previously], but that’s difficult for me to do now…[chlorinated water] has a taste that is not normal. With this [WAPI], it [the water] has no taste at all. [Peri-urban woman, age 31, purchased drinking water from cart with small drums]

Participants often mentioned that the short amount of time it took to treat water was an advantage of using the WAPI and noted that the time needed for WAPI use compared favorably with boiling or treating water with chlorine or bleach. As one participant compared WAPI use with boiling:

The advantage [of the WAPI] is that it helps me save time. It’s a savings that I’ve noted with [the WAPI]. That’s the advantage it gives me, because I don’t wait 10 to 15 minutes to be sure the water is safe anymore. With this, I’m good [to go] sooner. [Peri-urban woman, age 37, purchased bottled water]

Another participant compared the time of WAPI use with that of chlorination:

I used to treat [my water] with chlorine, and that took a while and had a [bad] taste. Now that I’m treating [my water] with the indicator, it’s good and it doesn’t taste like anything and it’s easy and it’s fast. [Peri-urban woman, age 31, purchased drinking water from cart with small drums]

At the habitual level, participants frequently noted that both models were easy to use. For those participants who reported difficulty using the models, the most commonly reported difficulty was confusion in how to use the model at the start of the trial, which was typically resolved by referring to the WAPI’s accompanying instructions. Participants frequently justified any preference they had of one model over the other by citing ease of use, suggesting that this was the main factor affecting the acceptability of a WAPI design. Some participants mentioned specific characteristics of the models that affected how easy they were to use. For example, some participants noted that the wire model was difficult to manipulate, particularly when the wire was heated by contact with the pot. One participant mentioned that the tube of the wire WAPI model could shift to an angled or horizontal position, which could cause the wax to harden along a vertical edge of the tube and the WAPI would need to be reheated and the wax hardened at the end of the tube to use the WAPI again. Another participant reported that the wire model was difficult to use without having a pot with a handle to which the wire could be tied.

Participants frequently compared the ease of use of the WAPI models with that of other treatment methods. In comparison with chlorination, participants frequently stated that using the WAPI was easier because it took less time and they felt greater self-efficacy using the WAPI. A participant stated:

I liked [the indicator] because it’s easier to treat water in comparison with chlorine…it’s more comfortable…because [with chlorine] I might do it wrong. Might treat it wrong, you know? More than anything, it’s because of that. With this, I feel more comfortable. [Rural woman, age 19, purchased drinking water from tanker truck]

As compared to boiling, some participants stated that WAPI use was easier because it took less time. One participant said,

It makes it easier because…it helps me, without having to wait for water to boil an hour, a half an hour. It’s more practical. One waits 10 minutes, 15 minutes, and the water’s ready.” [Rural woman, age 23, household piped drinking water user]

At the conclusion of both household trials, 77% (20/26) of the participants overall and 57% and 100% in the rural and peri-urban communities, respectively, stated a preference for the box model. A significantly smaller proportion of participants—15% (4/26) overall—preferred the wire model (*P* < 0.001), and 8% (2/26) had no preference.

## DISCUSSION

The findings of this study demonstrate the feasibility and acceptability of two WAPIs in rural and peri-urban communities in the Peruvian Amazon. The acceptability of this type of intervention was influenced by a variety of factors including ease of use, the time required to treat, the taste of treated water, knowledge of the relationship between water quality and health, self-efficacy to use the WAPI, literacy, gender roles, and perception of the effect of treatment on drinking water safety. Ease of use was a major factor influencing the acceptability of the interventions and the only factor that distinguished the two designs.

In the contextual dimension, this study was conducted in communities with a regional Ministry of Health policy of free chlorine production and low-cost availability of commercial bleach products, which likely facilitated the participants’ prior experience using chlorine or bleach to treat household drinking water. Although participants noted that WAPI use was easier and faster than either boiling or chlorine or bleach treatment, prior treatment with chlorine or bleach may have made enhanced positive perceptions of the taste of WAPI-treated water, resulting in greater acceptability of WAPI use in the study population. Community water sources may likewise have affected the acceptability of the WAPIs, particularly at the peri-urban site, where the primary community water source was vended water and secondary sources were frequently free of charge; thus, use of the WAPIs could facilitate cost savings while helping to ensure high-quality drinking water. At the household level, the WAPIs were frequently used by the female household member responsible for the management of household drinking water, suggesting that the study communities were favorable environments for habitual WAPI use. However, low literacy served as a barrier to adoption and consistent use for some participants. This finding is of concern because of evidence that Peruvian households headed by a person with less than a primary school education are more likely to use unimproved or inadequately chlorinated water sources.^45^ Although nearly all participants who completed the study follow-up visits were able to correctly use both WAPI models, targeted promotion approaches or additional support to promote self-efficacy of WAPI use may help improve adoption of this intervention among potential users with low literacy.

On the psychosocial dimension, knowledge of the risk of waterborne disease transmission and aspirations to protect the health of family members motivated WAPI use. Although other Peruvian populations have demonstrated knowledge of the association between heat disinfection of water and health,^46–49^ low-income populations in other contexts have not demonstrated this knowledge.^50^ Our finding that family health motivated WAPI use conflicts with a previous study of a WAPI intervention in Peru,^30^ and evidence that health is a motivator of adoption of other HWT methods in Peru is mixed.^33,48,51,52^ This difference may be attributable to our use of local health post registers to recruit participants, as these individuals had likely already received education regarding the relationship between HWT and health and were actively engaged in healthcare seeking on behalf of their young child. Participants’ self-efficacy of WAPI use contributed to the acceptability of the WAPI intervention, and was greater than their self-efficacy to use chlorine, which has been previously noted as a barrier to chlorination in the region.^33^ Given that some behavioral theorists believe self-efficacy is the most important determinant of preventive health behaviors,^53^ this finding suggests that a WAPI may be an acceptable alternative HWT method in populations where considerable barriers to chlorination exist. The perception that WAPI use improved the safety of the water indicated that participants believed WAPI use was preferable to nontreatment, which was frequently reported at baseline. Water pasteurization indicator use thus represented a relative advantage over common practice, which is another key attribute motivating technology adoption.^54^

At the technology level, ease of use was a key factor that influenced the acceptability of both WAPIs and differentiated the two models. Ease of use was an important advantage of WAPI use in a previous study of the wire model used in this study^30^ and has been noted as important in influencing the adoption of interventions.^54^ Similarly to a previous WAPI study,^30^ the short period of time required for water treatment was a key factor that contributed to the acceptability of the WAPI. Participants noted that time required for treatment using the WAPI compared favorably with that of chlorination or boiling, suggesting that WAPI use may help in time saving for households that treated their water at baseline. Because most participants did not treat their water or did so inconsistently at baseline, time saving may not be perceived as an important benefit of WAPI use as compared with usual practice in this population. The taste of WAPI-treated water was also a factor affecting the acceptability of the interventions, and perceptions of taste at the individual level were more positive than at the household level. This difference may reflect social desirability bias, as the participant may have felt more comfortable sharing the negative perceptions of other household members with the interviewer than her own. Taste was not cited as a factor affecting the acceptability of a WAPI model in a previous study^30^; this difference may reflect differences in baseline HWT practices, as taste may be a more salient concern in a population where chlorination is more frequently practiced. Although the taste of boiled water has been compared favorably with that of water treated using other methods,^55^ it has still been identified as a barrier to boiling practice,^50^ and heating water with herbs, cumin, or other locally acceptable additives has been recommended to improve taste.^50^ In our study, adding lemon juice to drinking water and using treated water to making lemonade were culturally accepted practices used to improve the taste of WAPI-treated water. As noted by participants in the present study and those of a study of chlorination in the region,^33^ it may additionally be helpful to advise users of the need of a period of habituation to the taste of water treated with a WAPI.

Although the aim of this study was to investigate the factors affecting the feasibility and acceptability of WAPI designs, our data also provided some insight into the effectiveness of this WAPI intervention. We were unable to meaningfully compare the prevalence of *E. coli* contamination between source and stored water samples in the peri-urban community because of the small number of source samples collected; however, the lack of difference in the prevalence of contamination between source and stored WAPI-treated water samples in the rural community suggests that the intervention was not effective in reducing household drinking water contamination. This finding is similar to that of a previous randomized trial of the wire WAPI model on the coast of Peru, which found that a WAPI and safe storage intervention did not significantly reduce contamination,^30^ but differs from a study in Kenya in which the prevalence of drinking water free of coliform bacteria was significantly higher after the implementation of a WAPI intervention.^29^ The effectiveness of a WAPI intervention as a HWT method likely depends on a number of factors such as effectiveness of treatment and posttreatment contamination in the home, and further investigation of these factors is needed to maximize the effectiveness of this intervention.

This study has a number of limitations. First, we relied on self-report to assess WAPI use, which may have been overstated because of social desirability bias. Because we did not perform direct observations to assess WAPI use, we could not confirm whether use occurred; however, the substantial contamination of stored household drinking water suggests that either the WAPIs were used inconsistently or pasteurized water was recontaminated through unsafe handling and storage. Second, courtesy bias may have led participants to underreport aspects of the interventions that they disliked; thus, the actual acceptability of the interventions assessed here may be lower than that reported by participants in this study. Third, we did not systematically assess whether WAPI-treated water was drunk by household members, and therefore do not know if drinking WAPI-treated water was uniformly accepted by all members of the household. Fourth, the high acceptability of the WAPI interventions of the present study may not generalize to other settings because of the importance of factors operating in the local context. For example, in a context with greater turbidity of source water, a WAPI intervention may be less acceptable than treatment methods that reduce turbidity, such as flocculation–disinfection.^55^ In addition, we conducted this study during the rainy season, which may have increased reliance on rainwater as a water source and thus increased perceived need to treat source water and acceptability of WAPI use. More frequent use of water boiling during the rainy season has been noted in other parts of Peru.^48^ Last, we evaluated the WAPI under controlled conditions, and we have insufficient information to assess the long-term adoption or sustainability of this type of intervention.

Our study demonstrates the feasibility and acceptability of using WAPIs to treat household drinking water in low-income communities in the Peruvian Amazon. Attributes of the technology influencing ease of use were important to ensure feasibility and acceptability. In populations where knowledge of the association between HWT and health is not widespread, additional health education messaging may be needed to promote acceptability of a WAPI intervention. In this population, a WAPI with a plastic box that floated on the water’s surface was the most acceptable low-cost WAPI design trialed. Although we used the results of this research to inform the design of a WAPI intervention for a subsequent health impact study, research in other populations is needed to optimize the design of this type of intervention and assess its effect on waterborne disease and drinking water safety in resource-limited settings.

## Supplemental file

Supplemental materials
