# Supplementary material for: Assessment of the Feasibility and Acceptability of Using Water Pasteurization Indicators to Increase Access to Safe Drinking Water in the Peruvian Amazon
Source: Am J Trop Med Hyg. 2020 May 4;103(1):455–64. doi: 10.4269/ajtmh.18-0963 (PMC7356428; doi:10.4269/ajtmh.18-0963)
Supplement: Supplementary file 1 [file tpmd180963.SD1.pdf]

Supplemental Information

Evaluation of factors that influence water treatment behavior in low-income communities in the Peruvian Amazon

Household Screening

**Location and Date**		**Response**
S1	Town/Population Center	
S2	Address	
S3	Interview Date<br>(e.g. 01-JAN-2010)	|__|__| |__|__|__| |__|__|__|__| **day month year**
S4	Interviewer Code	|__|__|__|__|

Verification of Household Eligibility

**Eligibility Criterion**		**Response**
E1	How old is the person who manages the water your family drinks at home?	|__|__| **years**<br>Is the person 18 years or older?<br>Yes 1<br>No 0 **IF NO, END**
E2	Does a child under the age of five live in this house?	Yes 1<br>No 0

Participant Identification Number |__|__|__|

Evaluation of factors that influence water treatment behavior in low-income communities in the Peruvian Amazon

Household Sampling

Location and Date		Response
S1	Town/Population Center	
S2	Address	
S3	Interview Date<br>(e.g. 01-JAN-2010)	|__|__| |__|__|__| |__|__|__|__| **day** **month** **year**
S4	Interviewer Code	|__|__|__|__|

Verification of Participant Eligibility

Eligibility Criterion		Response
E1	Are you the person who manages the water your family drinks at home?	Yes 1<br>No 0 **IF NO, END**
E2	How old are you?	|__|__| **years**<br>Is the participant 18 years or older?<br>Yes 1<br>No 0 **IF NO, END**
E3	Does a child under the age of five live in this house?	Yes 1<br>No 0 **IF NO, END**<br>**IF YES, COMPLETE INFORMED CONSENT AND SURVEY**

Participant Identification Information

Consent and Name		Response
P1	Consent has been read and obtained	Yes 1<br>No 0 **IF NO, END**
P2	Family Surname(s)	
P3	First Name(s)	
P4	Contact phone number	

Follow Up Visit # _____ **Participant Identification Number** |__|__|__|

Date (DD – MMM) |__|__| |__|__|__|

Baseline Interview

1. Sex (1) Male (2) Female |__|

2. What is your date of birth? |__|__| |__|__|__| 19|__|__|
(Ex. 01-JAN-2013) DD MMM YY

If the participant does not know their date of birth, ask to see their DNI (national identity card). If the participant does not have a DNI and does not know their date of birth, write 99-999-1999 as the date.

3. How many years of education have you completed? |__|
(1) No formal education
(2) Primary school incomplete
(3) Primary school complete (grades 1-6)
(4) Secondary school incomplete
(5) Secondary school complete
(6) Some superior (technical school/university)
(7) Superior completed – technical
(8) Superior completed – university
(9) Refused

4. During the last 12 months, what has been your main occupation? |__|
(1) Government employee
(2) Nongovernment employee
(3) Self-employed
(4) Not working for pay
(5) Student
(6) Homemaker/caring for family
(7) Retired
(8) Unemployed (can work)
(9) Unemployed (unable to work)

5. How many people, including yourself, live in your household? |__|__|
A household is defined as a group os people who share the same living área living room).

Number of adults age 18 and over |__|__|
Number of children 5-17 |__|__|
Number of children 0–4 |__|__|

6. What is the main source of drinking water of your household? |__|__|

Piped water
(1) Inside the house
(2) In the yard

Natural water source
(7) Surface water
(8) Rainwater

Covered well (or with pump)
(3) Inside the house
(4) Public/comunal

Uncovered well
(5) Inside the house
(6) Public/comunal

Other Sources
(9) Tanker truck
(10) Bottled water
(11) Other (Specify) ___________

7. a. Do you use sources other than this one to get drinking water? (1/0/9) |__|

b. If Yes: Besides your main water source, what other source do you most often use? |__|__|

Piped water
(1) Inside the house
(2) In the yard

Covered well (or with pump)
(3) Inside the house
(4) Public/comunal

Uncovered well
(5) Inside the house
(6) Public/communal

Natural water source
(7) Surface water
(8) Rainwater

Other Sources
(9) Tanker truck
(10) Bottled water
(11) Other (Specify) ___________

8.a. Do you do anything to treat the water you use for drinking? (1/0/9) |__|

[If the answer to qu. 8a is "No", skip to Qu. 9.]

b. If Yes: Can you tell me how you usually treat the water you use for drinking? ________________________________

i. Have you used this method in the last week? (1/0/9) |__|
ii. Did you use this method to treat the water that is currently stored in your home? (1/0/9) |__|
iii. How often do you use this method? |__|

(1) Never (2) Rarely (3) Sometimes (4) Most of the time (5) Always

iv. Why do you treat your water? What kinds of things remind you to treat it?
____

____
____

v. Do you ever not treat the water you drink at home? (1/0/9) |__|

[If the answer to Qu. 8b.v. is "No", end interview.]

vi. Can you tell me why you sometimes do not treat the water you drink at home?

____
____
____

vii. What do you think would motivate you to treat it? Is there anything that you need to help you to treat it? If so, what?

____
____
____

9. If you never treat your water: a. why do you not treat it?

____
____
____

b. What do you think would motivate you to treat it? Is there anything that you need to help you to treat it? If so, what?

____
____
____

Follow Up Visit # _____ **Participant Identification Number** |__|__|__|

Date (DD – MMM) |__|__| |__|__|__|

Follow Up Interview

1. Describe your water treatment practices. Are you treating the water you drink? If yes, how and when?

____

____

____

2. a. How was your experience using the indicator? |__|

(1) It helped to treat your water
(2) It did not make water treatment easier or more difficult
(3) It made water treatment more difficult

b. Anything else?

____

____

____

3. Can you describe the advantages or disadvantages of using the indicator?

____

____

____

4. Are you using the indicator? _______

5. At what times do you use it most?

____

____

____

6. In your house, who uses the indicator most? Why does he/she use it most?

____

____

____

Follow Up Visit # _____ **Participant Identification Number** |__|__|__|

Date (DD – MMM) |__|__| |__|__|__|

7. Did your neighbors have any reaction/comment from seeing your indicator? Were they interested? Did they want one for themselves?

____

____

____

8. a. How did the indicator affect your water treatment? |__|

(1) It made it more difficult (2) It had no effect (3) It made it easier

b. Anything else?

____

____

____

9. a. What do you think of the indicator? |__|

(1) You like it (2) You do not like it nor dislike it (3) You dislike it

b. Anything else?

____

____

____

____

____

10. a. Have you had any problems with the indicator? ___________

b. If Yes: What problems have you had?

____

____

____

Visit # _____ **Participant Identification Number** |__|__|__|

Date (DD – MMM) |__|__| |__|__|__|

Water Sampling and Indicator Assessment
(Complete at the Enrollment visit, Follow-Up visit 2, and Final visit)

E. coli Testing

Take a water sample from the household water source and from the stored drinking water container. Write the number of the house on the sample and on the survey.

Indicator Measures (Complete at Follow-up visit 2 and final visit)

1. Do you have your water pasteurization indicator? Can you please show me where it is?

a. Is the water pasteurization indicator observed in the home? (1) Yes (0) No |__|

b. If in the home: How far is the water pasteurization indicator from the container in which water is boiled? |__|

(1) In the container
(2) <1m from the container
(3) Between 1-3m of the container
(4) >3m of the container or in a different room

Comments on indicator location/condition: ___________________________

c. Please show me how you use your water pasteurization indicator. You do not need to heat any water, just demonstrate and explain how you would use your indicator when heating water for drinking:

i. Does the participant place the indicator in the water while it is heating? (1) Yes (0) No |__|

ii. Does the participant place the indicator in the water in a position such that the wax is at the top of the tube? (1) Yes (0) No |__|

d. When you heat water for drinking, how do you know when to stop heating the water? [DO NOT READ THE CHOICES TO THE PARTICIPANT] |__|

(1) When the water starts boiling
(2) When the wax has fallen to the bottom of the indicator tube
(3) Other (Specify):_________________

2. a. Does the indicator need to be replaced? (1) Yes (0) No |__|

b. If Yes: Specify the reason: _____________________________________
